# miR-144-5p and miR-451a Inhibit the Growth of Cholangiocarcinoma Cells Through Decreasing the Expression of ST8SIA4

**DOI:** 10.3389/fonc.2020.563486

**Published:** 2021-01-14

**Authors:** Wan Fu, Guangcai Yu, Junnan Liang, Pan Fan, Keshuai Dong, Bixiang Zhang, Xiaoping Chen, Hong Zhu, Liang Chu

**Affiliations:** ^1^Hepatic Surgery Center, Tongji Hospital, Tongji Medical College, Huazhong University of Science and Technology, Wuhan, China; ^2^Hubei Provincial Key Laboratory of Hepatobiliary and Pancreatic Diseases, Tongji Hospital, Tongji Medical College, Huazhong University of Science and Technology, Wuhan, China; ^3^Institute of Organ Transplantation, Tongji Hospital, Tongji Medical College, Huazhong University of Science and Technology, Wuhan, China; ^4^Department of Medical Oncology, The First Affiliated Hospital of Soochow University, Suzhou, China

**Keywords:** cholangiocarcinoma, miR-144-5p, miR-451a, ST8SIA4, tumor suppressor

## Abstract

Accumulating evidences indicate that non-coding RNAs play crucial roles in the progression of an extensive range of carcinomas. This study aimed to investigate the action mechanism of miR-144-5p and miR-451a in cholangiocarcinoma. We found that miR-144-5p and miR-451a were significantly decreased in cholangiocarcinoma patient samples compared to the adjacent normal bile duct samples. The downregulation of these two miRNAs was correlated with a more advanced disease state of cholangiocarcinoma patients. Overexpression of miR-144-5p and miR-451a suppressed the proliferation, invasion and migration of cholangiocarcinoma cells *in vitro* and inhibited xenograft tumor growth. Knockdown of these two miRNAs had the opposite effects. miR-144-5p and miR-451a regulated the expression of ST8 alpha-N-acetyl-neuraminide alpha-2,8-sialyltransferase 4 (*ST8SIA4*), and presented a correlation with ST8SIA4 in patient samples. Overexpression of ST8SIA4 promoted the proliferation, invasion and migration of cholangiocarcinoma cells, and the changes were reversed by upregulating the expression of miR-144-5p and miR-451a. Our findings indicated that miR-144-5p and miR-451a displayed a tumor suppressor role through decreasing the expression of ST8SIA4 in cholangiocarcinoma.

## Introduction

Cholangiocarcinoma is a highly aggressive malignancy originating from the epithelial cells of the intra- and extrahepatic biliary ducts (1–3). Cholangiocarcinoma accounts for 10% to 20% of deaths related to primary hepatic carcinoma (4). Currently, the only treatment for cholangiocarcinoma is surgical resection of the tumor, and traditional chemotherapy and radiotherapy have little effect on improving the long-term survival of patients (5). However, therapeutic surgery is only available for early-, but not for advanced-stage patients and the 5-year survival rates of patients is still below 20%–40%, despite the combination of surgery and chemotherapy (6). Molecularly targeted therapy displays obvious advantage of controlling cancer cell proliferation, as well as preventing or delaying recurrence and metastasis (7). It has a wide range of applications with less adverse effects and can be highly targeted. Therefore, it is an urgent need to improve our understanding about the molecular pathogenesis of cholangiocarcinoma, which forms a basis for discovering diagnostic markers and new therapeutic targets.

In recent years, the function of non-coding RNAs in the occurrence and development of cancers has been increasingly recognized (8, 9). microRNAs (miRNAs) are a class of small single-stranded non-coding RNAs ranging in size from 19 to 25 nucleotides which act as important post-transcriptional regulators by directly binding to 3’ untranslated regions (3’ UTR) of the mRNAs of specific target genes, and thereby induce their translation impairment or degradation (10). An increasing number of studies indicate that miRNAs play crucial roles in regulating cell proliferation, apoptosis, metastasis and epithelial-to-mesenchymal transition (EMT) in diverse cancers (11–13). Gene therapies that use miRNAs might be an effective approach to blocking tumour progression. miRNAs such as let-7, which has been shown to negatively regulate the Ras oncogenes, and miR-15 and miR-16, which negatively regulate BCL2, are promising candidates for cancer treatment (14). Additionally, many studies have highlighted that miRNA clusters work more validly compared with unitary miRNAs in regulating the development of varied carcinoma types (15–18). It has been reported that clustered miRNAs, such as miR-144-5p and miR-451a, inhibited the proliferation of bladder carcinoma cells by reducing the expression of their oncogenic target genes. miR-144/451, a double cis-trans miRNA loci, locates on chromosome 17 which could encode miR-144-5p and miR-451a. miR-144/451 gene locus played a suppressive role in esophageal carcinoma as determined by principal component regression analysis (19). Downregulation of miR-144/451 cluster promoted the progression of B-lymphomagenesis through activating and sustaining c-Myc (20). Stretch-induced activation of AMP-activated protein kinase (AMPK) in vascular smooth muscle is in part regulated by reduced levels of miR-144/451 and that this effect may play a role in promoting contractile differentiation of smooth muscle cells (21). However, whether and how miR-144-5p and miR-451a take part in the development and progression of cholangiocarcinoma is unclear and requires further investigation.

Sialylation is a glycosyl modification pattern that affects the development and progression of cancer (22, 23). The glycans on the surface of most cells exhibit high levels of sialylation and often play roles in cell-cell or intercellular matrix interactions. Sialyltransferases (Sts) and sialidases are the main enzyme classes that control sialylation patterns. Sts transfer sialic acids from a donor substrate to final sites including a number of glycoprotein and glycolipid carbohydrate groups (24). Many studies have elaborated that different kinds of sialic acid transferase play important roles in cancer progression. ST8SIA2 increased the invasion capacity and chemosensitivity of liver cancer (25). Higher levels of ST3Gal III made ovarian carcinoma cells more sensitive to taxol (26). ST6GalNAcI expression was sufficient to reinforce the tumorigenesis of MDA-MB-231 cells (27). ST8SIA4 promoted the progression of acute myelocytic leukemia (AML) cells toward multidrug-resistant tumors (28). It has been reported that sialylation is involved in the development of 5-FU resistance and the sialylation inhibitor 3F-Sia can be used as a chemosensitizer for cholangiocarcinoma (29). However, the wider biological effects of Sts in cholangiocarcinoma has rarely been reported.

In the present study, we demonstrated that miR-144-5p and miR-451a were downregulated in cholangiocarcinoma patient samples. miR-144-5p and miR-451a overexpression inhibited proliferation, invasion and migration of cholangiocarcinoma cells by down-regulating ST8SIA4. ST8SIA4 reversed the miR-144-5p- and miR-451a- induced the growth defect of cholangiocarcinoma cells, and presented a correlation with the expression of these two microRNAs in patient samples. The newly discovered miR-144-5p/miR-451a-ST8SIA4 signaling axis might be of importance to understand the progression and metastasis of cholangiocarcinoma cells.

## Materials and Methods

### Patient Tissue Samples

Twenty-three cholangiocarcinoma tissues and paired normal bile duct tissues were collected (from December 2016 to December 2017) for qRT-PCR analysis of miR-144-5p, miR-451a and ST8SIA4, and western blot analysis of ST8SIA4. 75 cholangiocarcinoma tissues (from July 2014 to April 2018) plus the above mentioned 23 cholangiocarcinoma tissues were used for correlation analysis. The person who performed tests were blind to the patients’ information. All the patients underwent cholangiocarcinectomy at the Hepatic Surgery Center of Tongji Hospital of Huazhong University of Science and Technology (Wuhan, China). All patients were confirmed the diagnosis of cholangiocarcinoma by pathological analysis, and complete clinicopathological data were acquired. All research on human materials was approved by the Ethics Committee of Tongji Hospital, and the study was conducted according to the Declaration of Helsinki principles. Written informed consent was obtained from each patient.

### Cell Culture

The human cholangiocarcinoma cell lines HuCCT-1, HCCC-9810 and RBE were purchased from the RIKEN bioresource Center (Saitama-ken, Japan). The TFK-1 cell line was purchased from DSMZ (Deutsche Sammlung von Mikroorganismen und Zellkulturen GmbH, Braunschweig, Germany). Cells were cultured in Roswell Park Memorial Institute 1640 (RPMI 1640, Thermo Fisher Scientific, New York, USA) medium with 10% fetal bovine serum (FBS, Gibco, Thermo Fisher Scientific) at 37°C in a humidified atmosphere comprising 5% CO_2_. All cell lines were tested for mycoplasma contamination (Genecreate Biological engineering Co., Ltd, Wuhan, China).

### RNA Purification and qRT-PCR

Total RNA was isolated using the TRIzol reagent (Invitrogen, Carlsbad, CA, USA) according to the manufacturer’s instructions. First strand of mRNA was conducted with 2µg of total RNA, and real-time quantitative polymerase chain reaction was performed using FastKing gDNA Dispelling RT SuperMix Kit and Super Real Premix Plus Kit (SYBR Green) (Tiangen, Beijing, China. Cat.no.KR118&FP205), respectively. Poly(A)-tailing-based qRT-PCR methodology was applied to detect and measure the expression of miRNA using miRcute Plus miRNA First-Strand cDNA Kit and miRcute Plus miRNA qPCR Kit (SYBR Green) (Tiangen, Cat.no.KR211&FP411) with the CFX96 Real-time PCR system (Bio-Rad, Hercules, CA, USA). U6 was selected as the internal control for miRNA detection and GAPDH for mRNA. The expression level for each miRNA and mRNA was quantified using the 2^-△△Ct^ method. The following primers were used: U6 forward 5’-TCGCTTCGGCAGCACATATAC -3’, reverse 5’- GCGTGTCATCCTTGCGCAG -3’; miR-144-5p forward 5’- GGGGGGCATCATATACTGTAAG -3’; miR-451a forward 5’- GCCCCGTTACCATTACTGAGTT -3’; ST8SIA4 forward 5’- ATGCGCTCCATTAGGAAGAGG -3’, reverse 5’- GAGCTATTGACAAGTGACCGAC -3’; GAPDH forward 5’- CTCATGACCACAGTCCATGC -3’, reverse 5’- GGATGACCTTGCCCACAG -3’.

### Cell Transfection

Cells were cultured in six-well plates and transfected with miR-144-5p and miR-451a mimic/inhibitor or negative control (NC) (RiboBio, Guangzhou, China) at a final concentration of 50nm/100nm using Lipofectamine 2000 (Thermo Fisher Scientific) according to the manufacturer’s instructions. Cells were transfected with ST8SIA4 siRNA or NC (RiboBio) at a final concentration of 50 nm. The CDS sequence of ST8SIA4 was cloned into pcDNA3.1 plasmid (vigene Bioscience, Shandong, China) and transfected into cells to up-regulate ST8SIA4 expression.

### Xenograft Tumor Model

Five-week-old male BALB/c (nu/nu) mice were randomly divided into six groups (six mice each). According to the Guide for the Care and Use of Laboratory Animals, all of animals in the research were carefully cared and disposed. Experiments were approved by the Committee on the Ethics of Animal Experiments of Tongji Hospital of Huazhong University of Science and Technology (Wuhan, China). HCCC-9810 cells transfected with miR-144-5p, miR-451a mimics or inhibitors or controls were subcutaneously injected into the left flank of mice (1×10^7^ cells for mimics group; 5×10^6^ cells for inhibitor group). The tumor size was determined every 3 days by measuring the tumor diameter using a Vernier calipers, and calculated with the formula: Tumor volume (mm^3^) = d^2^ × D/2 (d and D represent the shortest and longest tumor diameter, respectively). All experimental mice were sacrificed 27 days after injection. All tumors were excised, weighed and fixed in 4% paraformaldehyde. Investigator was blinded to the group allocation during measuring tumor size, weight and performing immunohistochemical staining.

### Luciferase Reporter Assay

The wild and mutated binding sites between 3’ UTR of ST8SIA4 transcript 1 and miR-144-5p, 3’ UTR of ST8SIA4 transcript 2 and miR-451a, predicted by online database miRDB, were chemically synthesized by Tsingke biological technology Co. (Beijing, China) and were cloned into the XhoI/NotI sites of the psicheck-2 vector (Promega, Beijing, China). Cholangiocarcinoma cells were transfected with psicheck-2-ST8SIA4 and miR-144-5p, miR-451a or NC mimics using Lipofectamine 2000 (Invitrogen). The Dual-Luciferase Assay Kit (Promega) was used to measure the relative luciferase activity at 24 h after transfection.

### Transwell Assay

Transwell chambers (24-well insert, 8 mm pore size, Corning Costar, Cambridge, MA, USA) were used to assess the migration and invasion capacity of cholangiocarcinoma cells *in vitro*. For the cell migration and invasion assay, the lower chambers were filled with 650 µl of RPMI-1640 medium with 10% FBS. For the cell invasion assay, the chamber membrane was pre-coated with Matrigel (BD, Franklin Lakes, NJ, USA). The appropriate number of cells were resuspended in 100 µl of serum-free RPMI-1640 and seeded into the upper part of the transwell chambers. After incubation at 37°C (24 h for HuCCT-1, HCCC-9810 and RBE cells, 40 h for TFK1 cells), cells were fixed and stained using 0.4% paraformaldehyde and 0.1% crystal violet, respectively, and Image-Pro Plus (IPP) v6.0 software (Media Cybernetics Inc., Rockville, MD, USA) was used to count the migrating/invading cells. The experiments were performed in triplicate.

### CCK-8 Assay

Cells were seeded into a 96-well plate. Cell viability was analyzed using Cell Counting Kit-8 (CCK-8; Dojindo, Kumamoto, Japan) according to the manufacturer’s instructions.

### Western Blot Analysis

Total proteins were extracted from cholangiocarcinoma tissues and cells, and protein concentrations were measured using the BCA protein assay kit (Thermo Fisher Scientific). Protein extracts were separated on 10% SDS-PAGE gels and subsequently transferred to PVDF membranes. The membranes were incubated with antibodies against GAPDH (1: 10000; Abcam, UK) and ST8SIA4 (1:1000; ABclonal, USA) at 4°C overnight. Peroxidase-conjugated anti-mouse or anti-rabbit IgG (1: 5000; Thermo Fisher Scientific) was used as the secondary antibody, and the bands were visualized using ECL reagent (Thermo Fisher Scientific). ST8SIA4 was normalized to GAPDH levels upon band intensity quantification through the Image Lab software.

### Statistical Analysis

Statistical analyses of all the experimental data were performed using GraphPad Prism software (GraphPad Inc, La Jolla, CA, USA) or SPSS 17.0 statistical software (IBM, Chicago, IL, USA). The Spearman correlation analysis was performed to analyze the association between miR-144-5p or miR-451a and ST8SIA4 expression. Relationship between miR-144-5p, miR-451a or ST8SIA4 expression and the clinicopathological characteristics was evaluated using χ2 test. Differences between two groups were evaluated using Student’s *t*-test or Wilcoxon test. All experiments were repeated three times, and results are presented as mean ± S.D. Differences with P < 0.05 were considered statistically significant.

## Results

### The Expression of miR-144-5p and miR-451a Was Decreased in Cholangiocarcinoma Tissues

**Figure 1:**
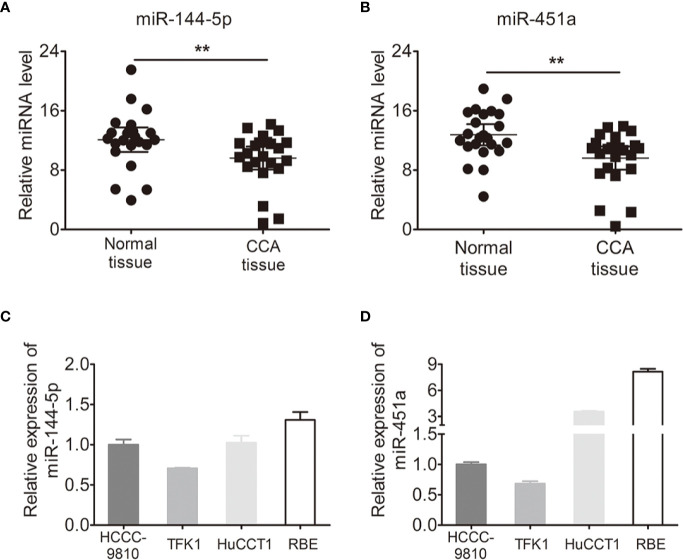
Expression of miR-144-5p and miR-451a in cholangiocarcinoma specimens and cell lines. **(A, B)** miR-144-5p **(A)** and miR-451a **(B)** expression levels in paired cholangiocarcinoma tissues and adjacent normal bile duct tissues detected by qRT-PCR (n=23). **(C, D)** miR-144-5p **(C)** and miR-451a **(D)** expression levels in cholangiocarcinoma cell lines relative to normal bile duct tissues analyzed by qRT-PCR. The data are shown as the mean ± S.D. **p < 0.01.

We used quantitative RT-PCR to identify the expression of miR-144-5p and miR-451a in 23 paired cholangiocarcinoma tissues and adjacent normal tissues. miR-144-5p and miR-451a levels were downregulated in cholangiocarcinoma tissues compared with normal tissues (**Figures 1A, B**). The association between miR-144-5p, miR-451a expression and the clinicopathological features of cholangiocarcinoma was assessed in 98 patient samples (**Table 1**). miR-144-5p and miR-451a expression levels were significantly correlated with HBsAg, tumor size and the presence of vascular invasion. Meanwhile, miR-451a expression level was correlated with tumor number. The downregulated expression of miR-144-5p and miR-451a suggested that they may play a tumor suppressor role in cholangiocarcinoma, and additional work will be required to confirm the expression and the association between expression and the clinicopathological features in more cholangiocarcinoma tissues. The endogenous expression of miR-144-5p and miR-451a was then examined in four cholangiocarcinoma cell lines. HCCC-9810 and TFK1 cells have low endogenous expression of these two miRNAs, and high endogenous expression was detected in HuCCT1 and RBE cells (**Figures 1C, D**).

**Table 1:** Correlations between miR-144-5p or miR-451a expression levels and clinicopathological features in cholangiocarcinoma.

Variables		miR-144-5 pexpression level		P-value	miR-451a expression level		P-value
		Low	High		Low	High	
Gender	Male	33	23	0.115	29	27	0.445
	Female	18	24		25	17	
Age(y)	<60	28	21	0.419	28	21	0.839
	≥60	23	26		26	23	
ALB(U/L)	<28	2	2	0.917	3	1	0.269
	28–35	8	6	0.917	10	4	0.255
	>35	41	39	0.826	41	39	0.121
HBsAg	No	21	33	**0.005**	24	30	**0.025**
	Yes	30	14		30	14	
HCVAg	No	49	46	1.000	51	44	0.250
	Yes	2	1		3	0	
Tumor number	Single	25	30	0.158	24	31	**0.014**
	Multiple	26	17		30	13	
Tumor size	<5cm	15	27	**0.008**	15	27	**0.001**
	≥5cm	36	20		39	17	
TNM stage	I–III	40	41	0.294	48	33	0.107
	IV	11	6		6	11	
Vascular invasion	No	22	30	**0.046**	22	30	**0.008**
	Yes	29	17		32	14	
LN metastasis	No	32	30	1.000	37	25	0.293
	Yes	19	17		17	19	

The chi-square tests were adopted to analyze correlation between miR-144-5p or miR-451a levels and clinicopathological features in cholangiocarcinoma. The mean value of expression level was used as the cutoff. Bold values had statistical significance. intrahepatic cholangiocarcinoma=84, perihilar cholangiocarcinoma=14.

### miR-144-5p and miR-451a Regulated the Proliferation, Invasion, and Migration of Cholangiocarcinoma Cells *In Vitro*

**Figure 2:**
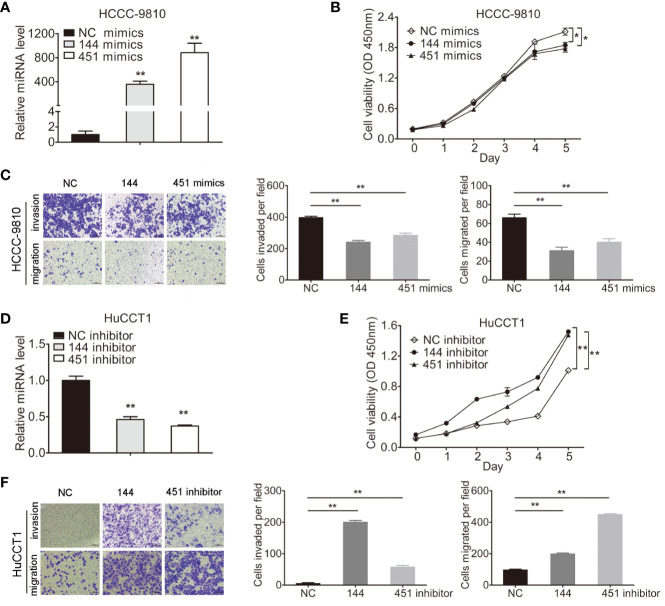
miR-144-5p and miR-451a altered the proliferation, invasion and migration of cholangiocarcinoma cells *in vitro*. **(A, B)** Using miR-144-5p and miR-451a mimics or inhibitor to overexpress **(A)** or downregulate **(B)** the expression of miR-144-5p and miR-451a. **(C, D)** Overexpression of miR-144-5p or miR-451a in HCCC-9810 and TFK1 cells suppressed **(C)** cell proliferation measured by CCK8, **(D)** cell invasion and migration detected by matrigel-coated transwell and transwell assay. **(E, F)** Downregulating the expression of miR-144-5p or miR-451a in HuCCT1 and RBE cells promoted **(E)** cell proliferation, **(F)** cell invasion and migration as measured as **(C, D)**. All experiments were repeated 3 times. Bars represent mean ± S.D. *p < 0.05. **p < 0.01.

miR-144-5p and miR-451a mimics or inhibitors were transfected into four cholangiocarcinoma cell lines according to their endogenous expression, respectively (**Figures 2A, D**; **Supplementary Figures S1A, D**). Overexpression of miR-144-5p or miR-451a significantly reduced cell proliferation, invasion and migration abilities in HCCC-9810 (**Figures 2B, C**) and TFK1 cells (**Supplementary Figures S1B, C**). Conversely, the opposite effects on cell proliferation, invasion, and migration were observed when miR-144-5p and miR-451a were knocked down in HuCCT1 (**Figures 2E, F**) and RBE cells (**Supplementary Figures S1E, F**).

### miR-144-5p and miR-451a Had Effect on Tumor Growth *In Vivo*

**Figure 3:**
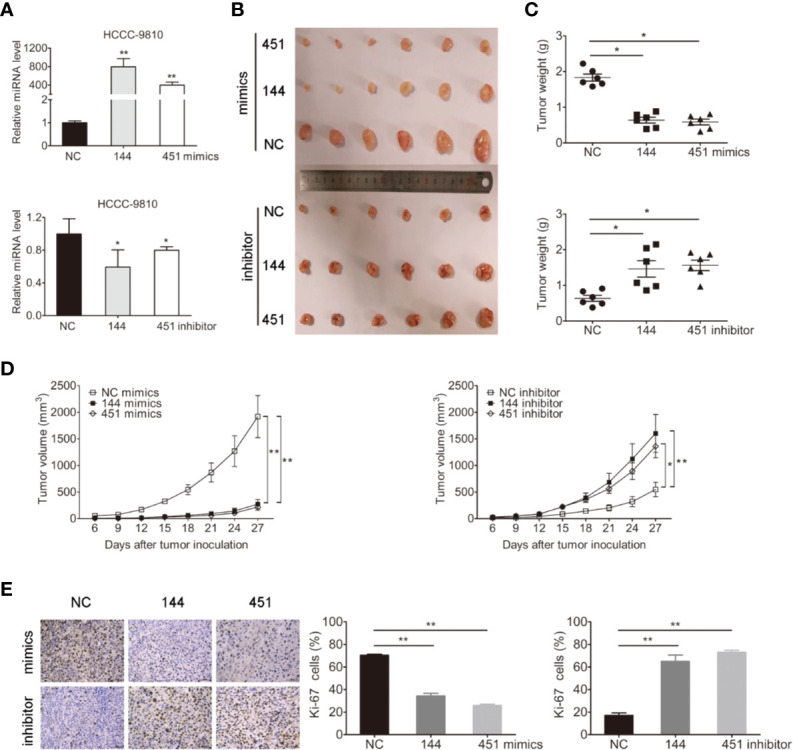
miR-144-5p and miR-451a changed tumor growth *in vivo*. **(A)** Using miR-144-5p and miR-451a mimics or inhibitor to overexpress or downregulate the expression of miRNA-144-5p and miRNA-451a in HCCC-9810 cells. **(B)** Representative images of xenograft tumors derived from miR-144-5p or miR-451a overexpressed- or knockeddown-HCCC-9810 cells. Mice were sacrificed 27 days after subcutaneous inoculation. **(C)** Tumor weight of xenografts. **(D)** Tumor volumes were monitored every 3 days. **(E)** Representative immunohistochemistry staining images of Ki-67 in the indicated tumor tissues, and quantification of Ki-67 positive cells. Bars represent mean ± S.D. (n = 6), *p < 0.05. **p < 0.01.

We next evaluated whether miR-144-5p and miR-451a mediated tumorigenesis *in vivo*. Because TFK1, HuCCT1, and RBE cells cannot form subcutaneous tumors, HCCC-9810 cells with overexpression or downregulation of miR-144-5p or miR-451a were subcutaneously injected into nude mice, respectively (**Figure 3A**). Each tumor was measured every 3 days and weighted after all mice were sacrificed. Both the average weight and volume of tumor xenografts were markedly suppressed by the overexpression of miR-144-5p or miR-451a compared with the control groups. Knockdown of miR-144-5p or miR-451a had the opposite effects (**Figures 3B–D**). Additionally, immunohistochemical staining for the proliferation marker Ki-67 was used to reveal changes of tumor proliferation. Tumors with miR-144-5p or miR-451a overexpression had lower Ki-67-positive cells. Down-regulating miR-144-5p and miR-451a expression increased the Ki-67-positive cells (**Figure 3E**).

### miR-144-5p and miR-451a Regulated the Expression of ST8SIA4

**Figure 4:**
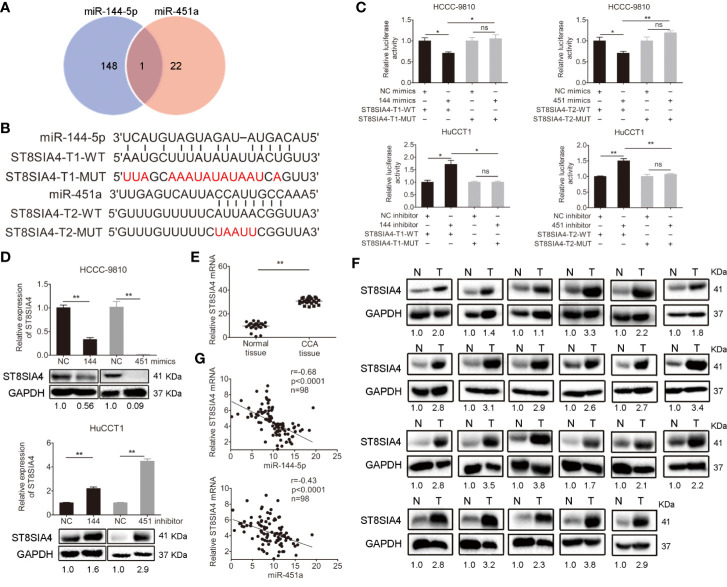
Validation of ST8SIA4 as a direct target of miR-144-5p and miR-451a. **(A)** The Venn diagram displays the potential targets of miR-144-5p and miR-451a. **(B)** The sequences of the potential binding sites between 3’ UTR of ST8SIA4 (ST8SIA4-T1-WT, ST8SIA4-T2-WT) and miR-144-5p, miR-451a. ST8SIA4-T1-MUT and ST8SIA4-T2-MUT are the binding sites-mutated segments. **(C)** Luciferase activities were measured 24 h after the indicated cells transfected with miR-144-5p, miR-451a mimics or inhibitor and reporter plasmid containing wild type 3’ UTR regions of ST8SIA4 or mutated segments. **(D)** ST8SIA4 mRNA and protein expression levels in the indicated cholangiocarcinoma cells transfected with miR-144-5p, miR-451a mimics or inhibitor. **(E, F)** ST8SIA4 mRNA **(E)** and protein **(F)** expression levels in paired cholangiocarcinoma tissues and adjacent normal bile duct tissues (n=23) examined by qRT-PCR and western blot, respectively. N, normal tissue. T, CCA tissue. **(G)** Linear regression analysis of the correlation between ST8SIA4 and miR-144-5p or miR-451a in patient samples (n=98). All experiments were repeated 3 times. Bars represent mean ± S.D. *p < 0.05. **p < 0.01. n.s, non-significant.

The miRDB, PicTar TargetScan, and miRanda algorithms were chosen to predict the potential targets of miR-144-5p and miR-451a. There were 148 and 22 predictive targets of miR-144-5p and miR-451a, respectively, and only one common candidate target, which is the *Homo sapiens* ST8 alpha-N-acetyl-neuraminide alpha-2,8-sialyltransferase 4 gene (*ST8SIA4*, **Figure 4A**). ST8SIA4 has four transcripts and the potential binding sequences of miR-144-5p and miR-451a are located in the 3’ UTR of transcripts 1 and 2 (abbreviated as T1 and T2, **Figure 4B**). Luciferase reporter assay was performed to validate whether ST8SIA4 was a direct target of miR-144-5p and miR-451a. The miR-144-5p and miR-451a mimics significantly downregulated the luciferase activity of reporter vectors containing wild-type binding sequences. Whereas, mimics had no effect on the luciferase activity if the corresponding binding motifs were mutated. The opposite results were observed when cells were transfected with miR-144-5p and miR-451a inhibitor (**Figure 4C**; **Supplementary Figure S2A**). Both the mRNA and protein expression levels of ST8SIA4 were significantly decreased in HCCC-9810 and TFK1 cells transfected with miR-144-5p or miR-451a mimics, while the inhibitors of miR-144-5p or miR-451a upregulated the expression of ST8SIA4 in HuCCT1 and RBE cells (**Figure 4D**; **Supplementary Figure S2B**). Moreover, the transcriptional and translational levels of ST8SIA4 were increased to a certain extent in cholangiocarcinoma tissues compared with the adjacent normal bile duct tissues (**Figures 4E, F**). The association between ST8SIA4 expression and the clinicopathological features of cholangiocarcinoma was assessed in 98 patient samples (**Table 2**). ST8SIA4 expression levels were significantly correlated with HBsAg and the presence of lymph node metastasis. As expected, the expression of ST8SIA4 was correlated with the expression of miR-144-5p and miR-451a in patient samples (**Figure 4G**).

**Table 2:** Correlations between ST8SIA4 expression levels and clinicopathological features in cholangiocarcinoma.

Variables		ST8SIA4 expression level		P-value
		Low	High	
Gender	Male	35	21	0.559
	Female	26	16	
Age(y)	<60	31	18	0.500
	≥60	30	19	
ALB(U/L)	<28	2	2	0.219
	28–35	6	8	0.228
	>35	53	27	0.130
HBsAg	No	29	25	**0.042**
	Yes	32	12	
HCVAg	No	58	37	0.237
	Yes	3	0	
Tumor number	Single	32	23	0.234
	Multiple	29	14	
Tumor size	<5cm	25	17	0.393
	≥5cm	36	20	
TNM stage	I–III	53	28	0.127
	IV	8	9	
Vascular invasion	No	33	19	0.478
	Yes	28	18	
LN metastasis	No	44	18	**0.030**
	Yes	17	19	

The chi-square tests were adopted to analyze correlation between ST8SIA4 levels and clinicopathological features in cholangiocarcinoma. The mean value of expression level was used as the cutoff. Bold values had statistical significance. intrahepatic cholangiocarcinoma=84, perihilar cholangiocarcinoma=14.

### ST8SIA4 Promoted the Proliferation, Invasion, and Migration of Cholangiocarcinoma Cells *In Vitro*

**Figure 5:**
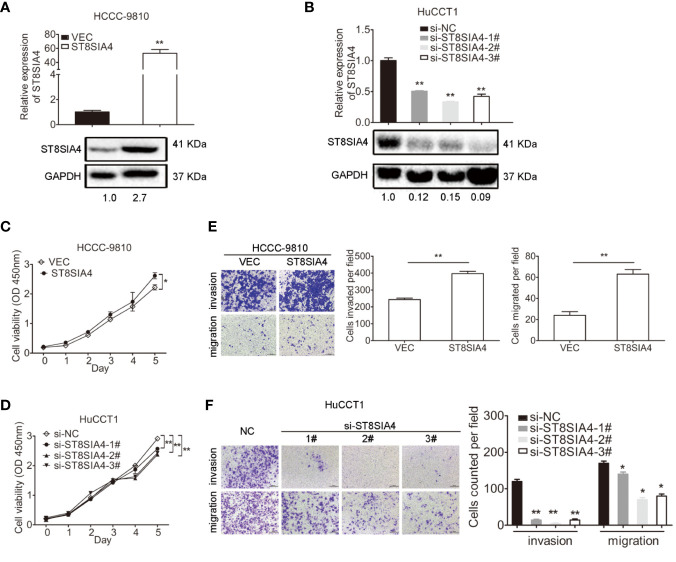
ST8SIA4 had effect on the proliferation, invasion and migration of cholangiocarcinoma cells *in vitro*. **(A)** Overexpression of ST8SIA4 in HCCC-9810 and TFK1 cells was confirmed by qRT-PCR and western blot. **(B)** Knockdown of ST8SIA4 in HuCCT1 and RBE cells was confirmed by qRT-PCR and western blot. **(C)** Overexpression of ST8SIA4 promoted proliferation of HCCC-9810 and TFK1 cells measured by CCK8 assay. **(D)** Overexpression of ST8SIA4 increased the invasion and migration of HCCC-9810 and TFK1 cells detected by matrigel-coated transwell and transwell assay. **(E)** Knockdown of ST8SIA4 decreased proliferation of HuCCT1 and RBE cells measured by CCK8 assay. **(F)** Knockdown of ST8SIA4 suppressed the invasion and migration of HuCCT1 and RBE cells as measured as **(D)**. All experiments were repeated 3 times. Bars represent mean ± S.D. *p < 0.05. **p < 0.01.

We next investigated the function of ST8SIA4 in cholangiocarcinoma. ST8SIA4 T1 and T2 have the same CDS sequence, and was cloned into the pcDNA3.1 vector to overexpress ST8SIA4 (**Figure 5A**; **Supplementary Figure S3A**). siRNA was used to downregulate its expression (**Figure 5B**; **Supplementary Figure S3B**). CCK8, transwell-matrigel and transwell assays were performed to assess the effects of ST8SIA4 on the proliferation, invasion and migration capacities of cholangiocarcinoma cells. Overexpression of ST8SIA4 significantly promoted these capacities of HCCC-9810 (**Figures 5C, E**) and TFK1 **(****Supplementary Figures S3C, E**) cells. Downregulation of ST8SIA4 significantly suppressed cell proliferation, invasion and migration of HuCCT1 (**Figures 5D, F**) and RBE (**Supplementary Figures S3D, F**) cells.

### miR-144-5p and miR-451a Restrained the Proliferation, Invasion, and Migration of Cholangiocarcinoma Cells by Regulating ST8SIA4

**Figure 6:**
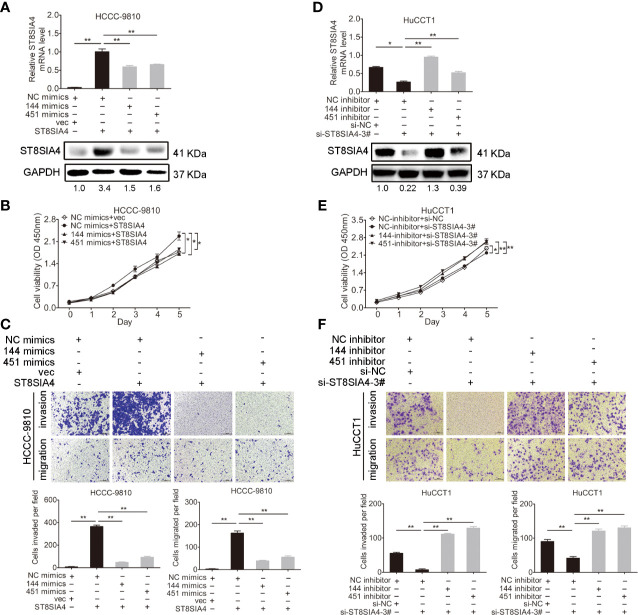
miR-144-5p and miR-451a regulated the proliferation, invasion and migration of cholangiocarcinoma cells through ST8SIA4. **(A)** The mRNA and protein expression of ST8SIA4 in HCCC-9810 and TFK1 cells co-transfected with the indicated miR-144-5p, miR-451a mimics, and plasmid containing ST8SIA4 was detected by qRT-PCR and western blot. **(B)** Cell proliferation of HCCC-9810 and TFK1 cells with the indicated co-transfection. **(C)** Cell migration and invasion of HCCC-9810 and TFK1 cells with the indicated co-transfection. **(D)** ST8SIA4 mRNA and protein expression in HuCCT1 and RBE cells co-transfected with miR-144-5p, miR-451a inhibitor and ST8SIA4 siRNA-3#. **(E)** Cell proliferation of HuCCT1 and RBE cells with the indicated co-transfection. **(F)** Cell migration and invasion of HuCCT1 and RBE cells with the indicated co-transfection. All experiments were repeated 3 times. Bars represent mean ± S.D. *p < 0.05. **p < 0.01.

**Figure 7:**
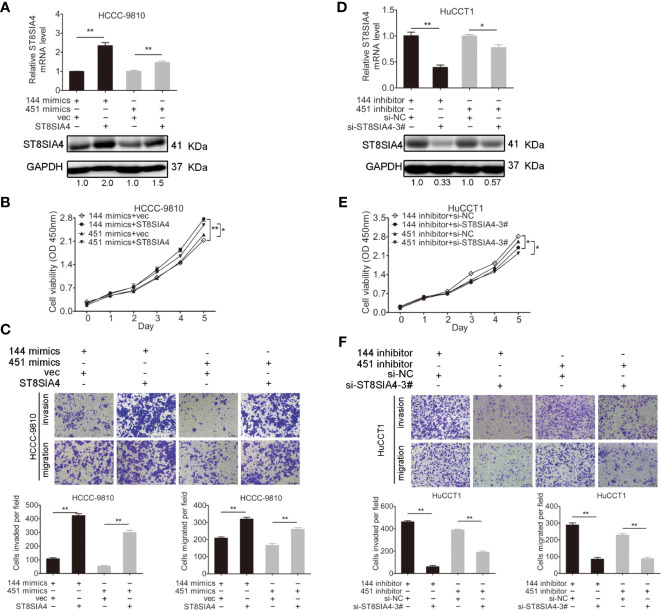
ST8SIA4 rescued the miR-144-5p- and miR-451a-induced the growth defect of cholangiocarcinoma cells. **(A)** The mRNA and protein expression of ST8SIA4 in HCCC-9810 and TFK1 cells co-transfected with the indicated miR-144-5p, miR-451a mimics and plasmid containing ST8SIA4 was detected by qRT-PCR and western blot. **(B)** Cell proliferation of HCCC-9810 and TFK1 cells with the indicated co-transfection. **(C)** Cell migration and invasion of HCCC-9810 and TFK1 cells with the indicated co-transfection. **(D)** ST8SIA4 mRNA and protein expression in HuCCT1 and RBE cells co-transfected with miR-144-5p, miR-451a inhibitor and ST8SIA4 siRNA-3#. **(E)** Cell proliferation of HuCCT1 and RBE cells with the indicated co-transfection. **(F)** Cell migration and invasion of HuCCT1 and RBE cells with the indicated co-transfection. All experiments were repeated 3 times. Bars represent mean ± S.D. *p < 0.05. **p < 0.01.

Cholangiocarcinoma cells were transfected with miR-144-5p, miR-451a mimics or inhibitors together with ST8SIA4 expression plasmids or siRNA-3# to further assess whether miR-144-5p and miR-451a can restrain the cholangiocarcinoma cell proliferation, invasion, and migration by regulating ST8SIA4. The increased cell proliferation, invasion and migration stimulated by upregulation of ST8SIA4 in HCCC-9810 (**Figures 6A–C**) and TFK1 (**Supplementary Figures S4A–C**) cells were notably suppressed by enhancing miR-144-5p and miR-451a expression. Conversely, downregulation of ST8SIA4 prevented HuCCT1 (**Figures 6D–F**) and RBE (**Supplementary Figures S4D–F**) cells from proliferating, invading and migrating, and all the effects were significantly suppressed by downregulating miR-144-5p and miR-451a. Moreover, ST8SIA4 rescued the miR-144-5p- and miR-451a-induced the growth defect of cholangiocarcinoma cells (**Figure 7**; **Supplementary Figure S5**).

## Discussion

In recent decades, radical surgery, chemotherapy and radiotherapy, which are comprehensive therapies, have been used to treat cholangiocarcinoma. However, the prognosis of patients with cholangiocarcinoma remains poor due to recurrence and metastasis of the neoplasm (30, 31). Therefore, it is urgent to understand the molecular mechanisms that drive the initiation and progression of cholangiocarcinoma during the early stages.

The overwhelming majority of confirmed miRNA clusters are regarded as independent units after transcription and usually originate from polycistronic mRNA sequences (32, 33). Several studies have revealed the downregulation of miR-144-5p and miR-451a expression in certain types of tumors, including colorectal and hepatocellular carcinoma (HCC), which suggested the cluster function as a potential cancer suppressor (34, 35). However, there are also studies with contradictory results. For example, Zhang et al. indicated that miR-144-5p accelerated the proliferation, migration and invasion of nasopharyngeal cancer cells (36). miR-144/451 regulated human epithelial cancer metastasis by suppressing the a disintegrin and metalloproteinase (ADAM) protein family members ADAMTS5 and ADAM10’s expression (37). miR-144/451 cluster inhibited esophageal cancer cell invasion through downregulating c-Myc and p-ERK (38). Overexpression of miR-144/451 could activate PTEN/AKT pathway to promote cell proliferation of insulinomas (39). Hence, the molecular mechanism by which the miRNA clusters act in tumorigenesis appears complex and extremely tissue-specific. We identified the expression of miR-144-5p and miR-451a was lower in cholangiocarcinoma tissues compared with normal tissues, and their expression levels were significantly correlated with HBsAg as well as tumor size, easier vascular invasion (**Figures 1A, B** and **Table 1**). It is known that miR-144/451 are associated to HCC with usually less than 15% of hepatitis B infection, which is the primary risk factor of hepatocellular carcinoma (HCC). In our cohort, frequency of hepatitis B is high compared to other studies, therefore, miR-144/451 may be related with only cholangiocarcinoma with hepatitis B. Our results demonstrated that miR-144-5p and miR-451a suppressed the proliferation, migration and invasion of cholangiocarcinoma cells (**Figure 2**; **Supplementary Figure S1**), which shed new light on the effect of non-coding RNA on cholangiocarcinoma development.

Sialic acids are negatively charged derivatives of the acidic sugar neuraminic acid and generally constitute the terminal ends of glycoconjugates. Sialyltransferases (Sts) are capable of catalyzing the transfer of sialic acid to proteins and lipids, and are involved in synthesizing the core structure of oligosaccharides (40). Changing the glycosylation patterns of glycoproteins generates variation and can accelerate cancerous transformation (41). Abnormal changes of Sts could lead to aberrant glycosylation, which is one of the significant hallmarks of carcinoma (42). It has been reported that ST8SIA4 is closely related to metastasis in breast cancer. Overexpression of ST8SIA4 promoted the invasion and migration of breast cancer cells, and the changes were reversed by upregulating the expression of miR-26a and miR-26b, which decreased the expression of ST8SIA4 (43). miR-146a and miR-146b promoted the progression of follicular thyroid carcinoma through targeting ST8SIA4 (44). In the present study, bioinformatics analysis revealed that ST8SIA4 was a potential target gene of both miR-144-5p and miR-451a (**Figures 4A, B**). Luciferase activity assays confirmed that miR-144-5p and miR-451a bound to the 3’ UTR of the ST8SIA4 mRNA (**Figures 4C, D**; **Supplementary Figure S2**). We also demonstrated that miR-144-5p and miR-451a inhibited the progression of cholangiocarcinoma through targeting ST8SIA4 (**Figure 6**; **Supplementary Figure S4**). ST8SIA4 has been identified as a potential glycan cancer marker in much of the literature (45). miR-181c was significantly decreased in drug-resistant chronic myelocytic leukemia (CML) cell lines and multidrug resistance (MDR) samples. Upregulation of miR-181c inhibited chemoresistance by targeting ST8SIA4 (46). Nevertheless, the mechanisms by which ST8SIA4 promoted the proliferation, migration and invasion capacities of cholangiocarcinoma cells remain to be elucidated.

Our data showed that ST8SIA4 was a direct target of miR-144-5p and miR-451a. The increased expression of miR-144-5p and miR-451a prevented proliferation, migration and invasion of cholangiocarcinoma cells through suppressing the expression of ST8SIA4. These findings demonstrated that miR-144-5p, miR-451a, and ST8SIA4 are functionally important components of cholangiocarcinoma, and may be promising targets in the future development of cholangiocarcinoma therapy.

## Data Availability Statement

The raw data supporting the conclusions of this article will be made available by the authors, without undue reservation.

## Ethics Statement

The studies involving human participants were reviewed and approved by the Ethics Committee of Tongji Hospital. The patients/participants provided their written informed consent to participate in this study. The animal study was reviewed and approved by the Committee on the Ethics of Animal Experiments of Tongji Hospital of Huazhong University of Science and Technology (Wuhan, China).

## Author Contributions

WF, HZ, and LC designed this study. LC supervised this study. WF, GY, and JL conducted the majority of the experiments. WF, GY, HZ, and LC analyzed the data and completed the manuscript. PF and KD participated in the experiments and the manuscript writing. BZ and XC revised the article critically for important intellectual content. All authors contributed to the article and approved the submitted version.

## Funding

This work was supported by grants from the National Natural Science Foundation of China (No. 31671348, 81871949, and 81572345), Chen Xiao-ping Foundation for the Development of Science and Technology of Hubei Province (CXPJJH12000001-2020317), and Jiangsu Six Talent Peaks Project (WSN-102).

## Conflict of Interest

The authors declare that the research was conducted in the absence of any commercial or financial relationships that could be construed as a potential conflict of interest.
