# Supplementary figures and images for: miR-144-5p and miR-451a Inhibit the Growth of Cholangiocarcinoma Cells Through Decreasing the Expression of ST8SIA4
Source: Front Oncol. 2021 Jan 14;10:563486. doi: 10.3389/fonc.2020.563486 (PMC7841262; doi:10.3389/fonc.2020.563486)

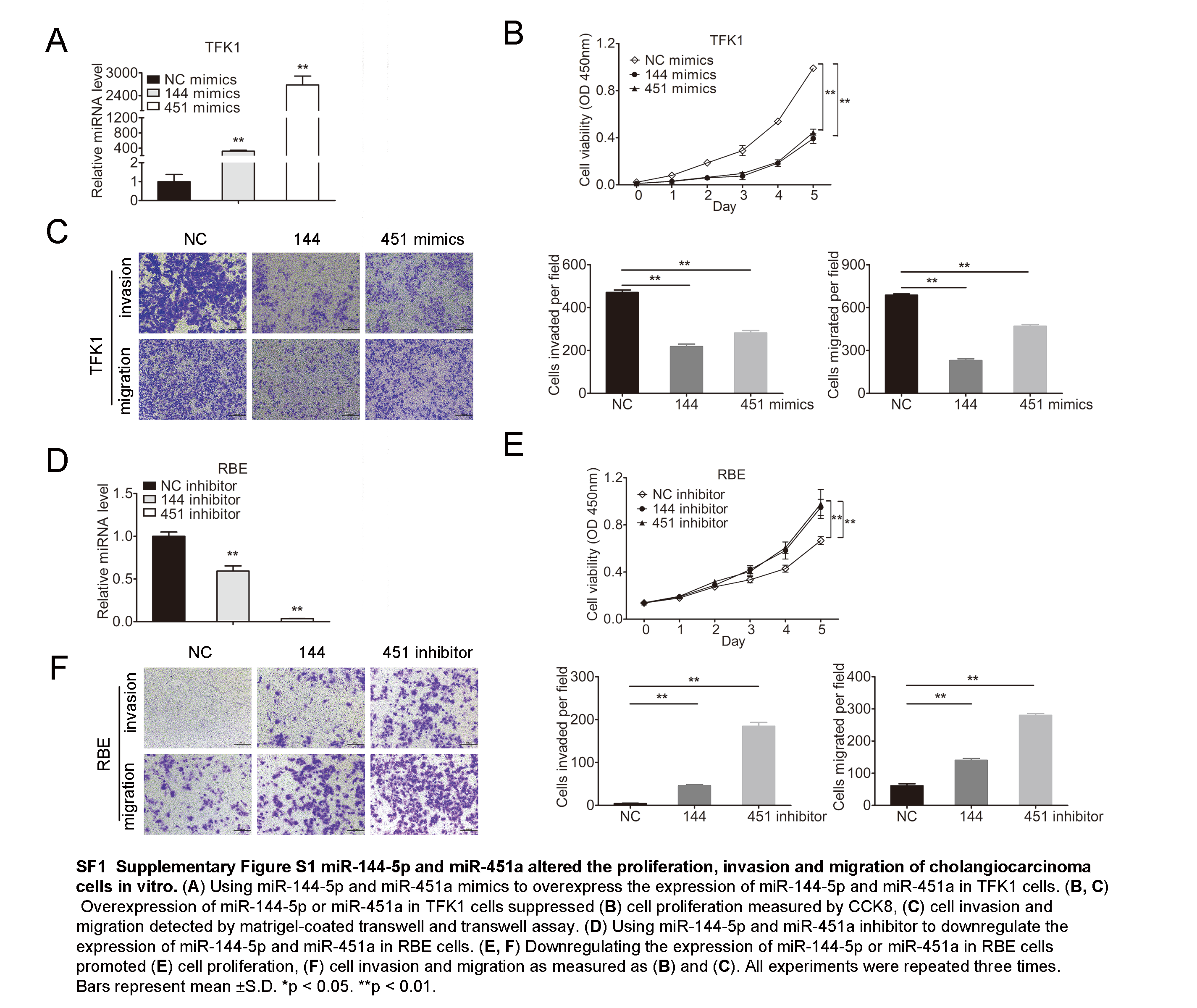

Supplement: Supplementary file 1 [file Image_1.tif]

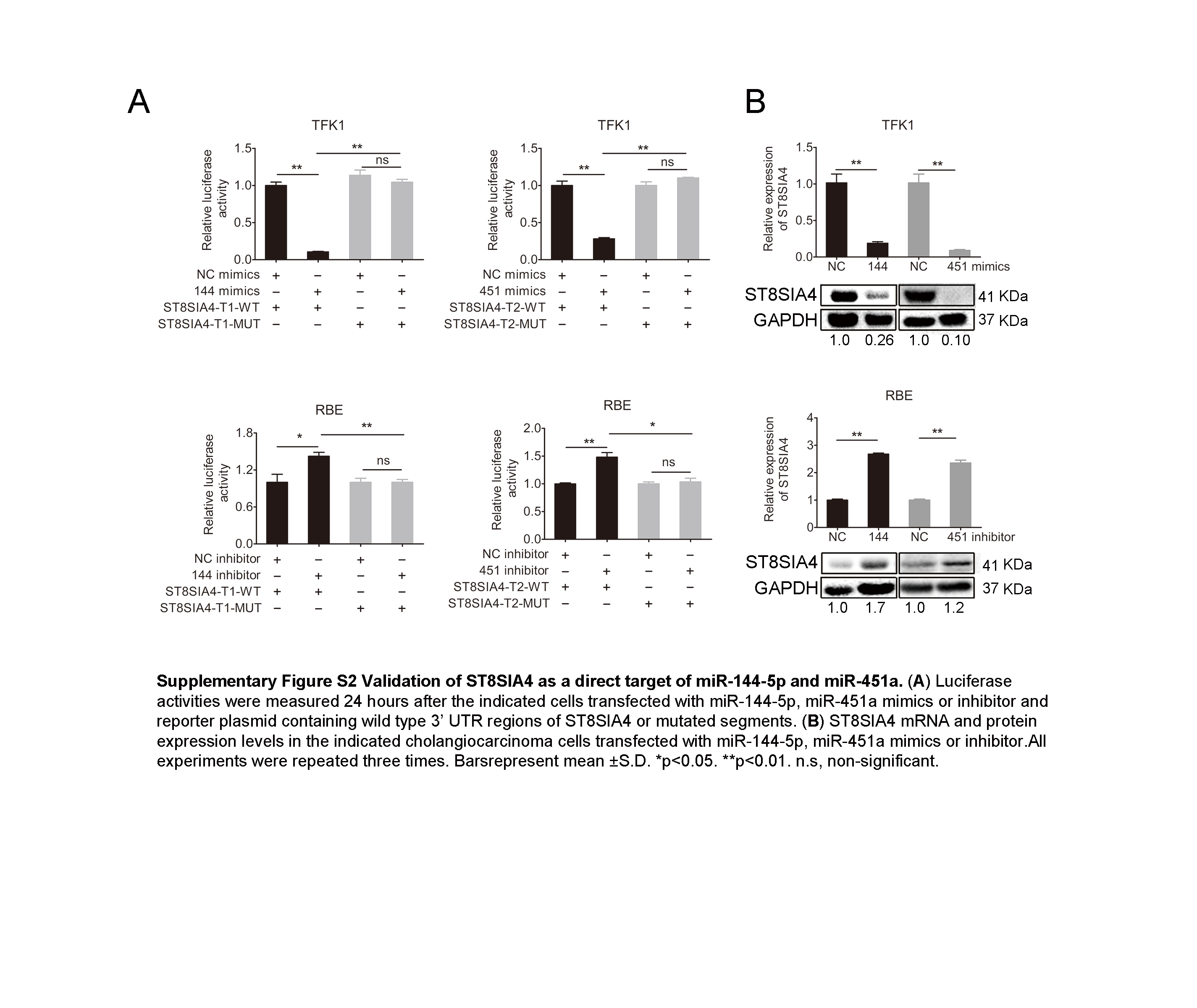

Supplement: Supplementary file 2 [file Image_2.tif]

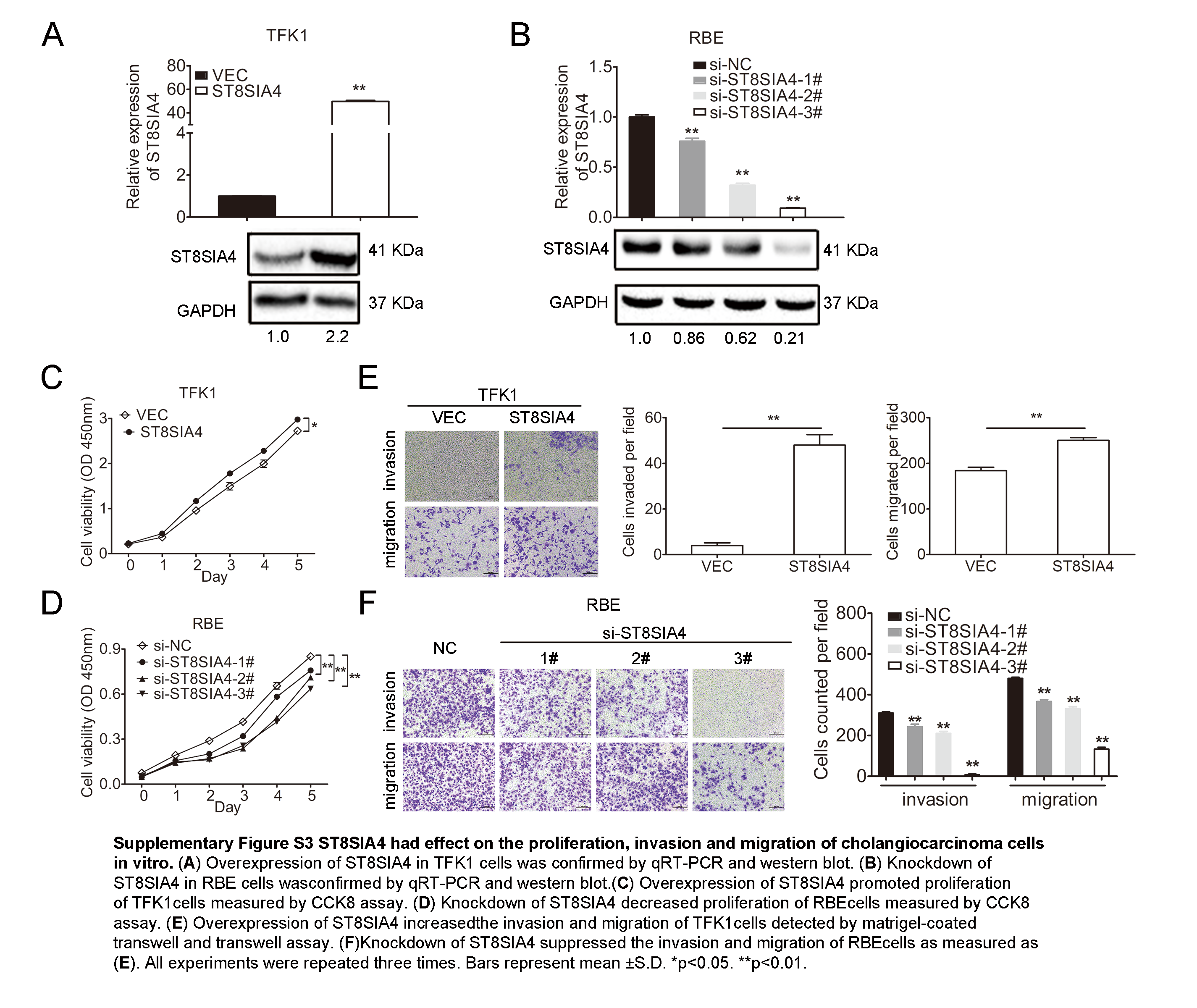

Supplement: Supplementary file 3 [file Image_3.tif]

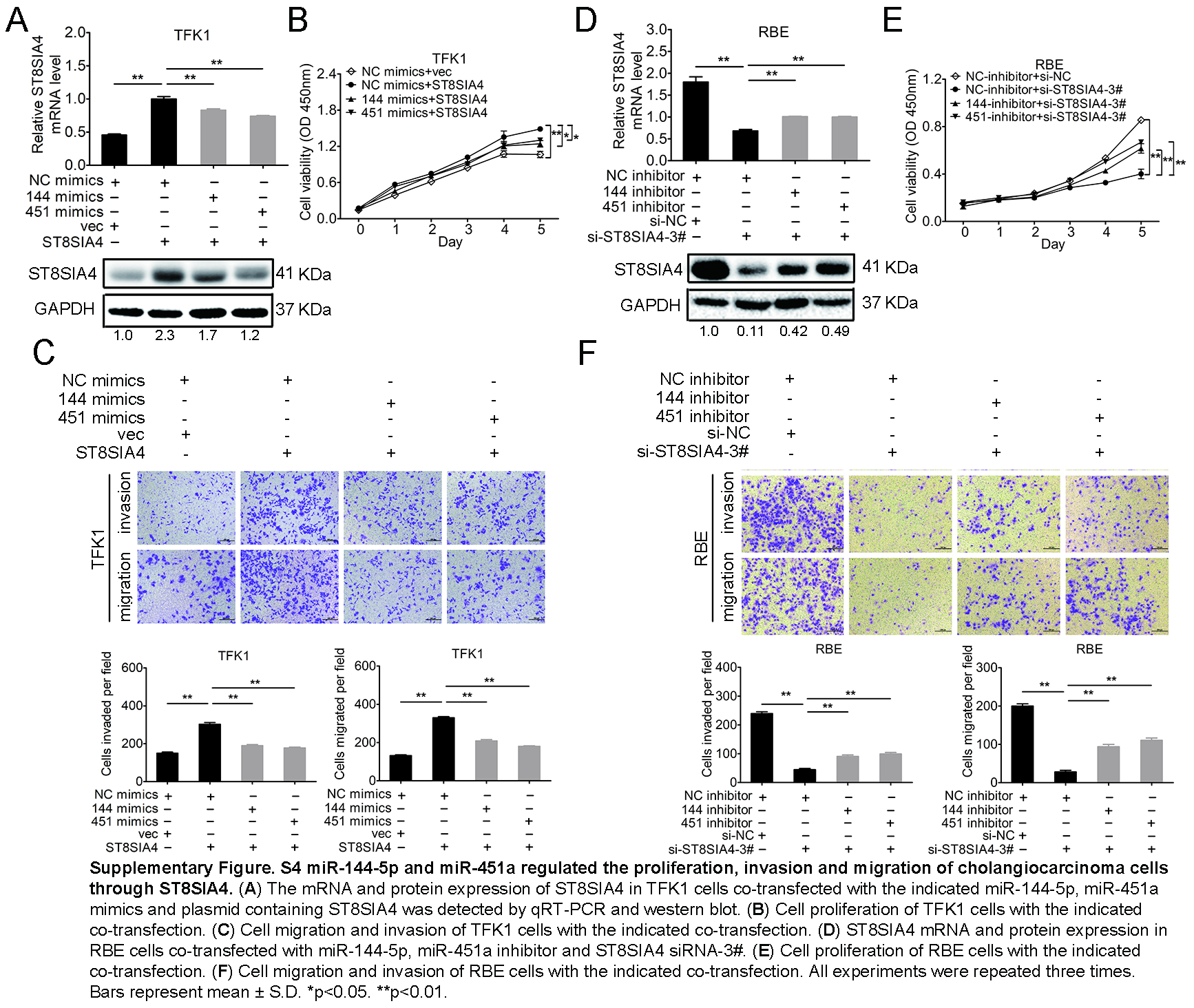

Supplement: Supplementary file 4 [file Image_4.tif]

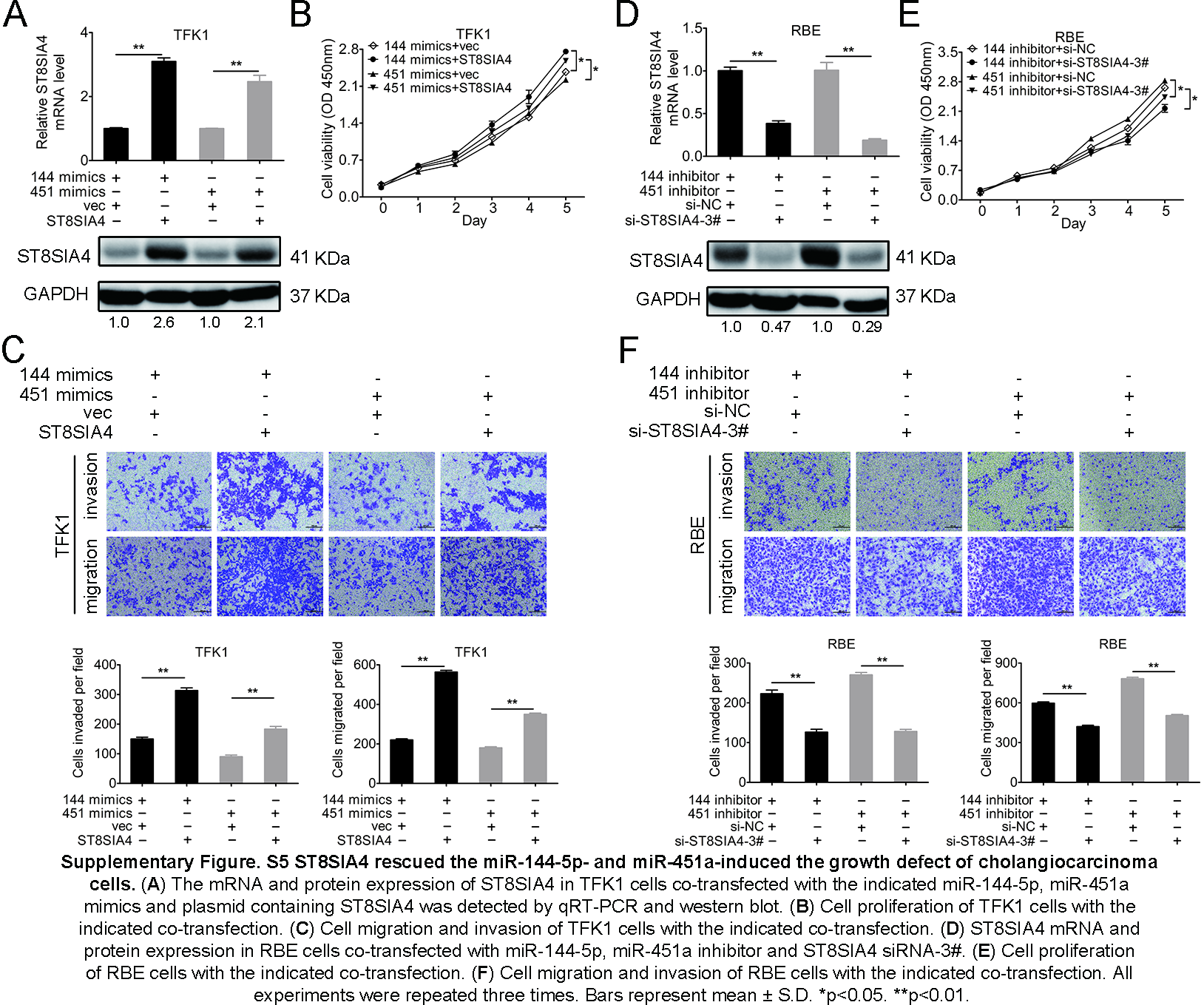

Supplement: Supplementary file 5 [file Image_5.tif]
